# Adverse Childhood Experiences and Flourishing Among Emerging Adult College Students: Religious Beliefs/Spirituality and Social Support as Research Domain Criteria (RDoC)-Informed Mechanisms

**DOI:** 10.1007/s10804-026-09560-2

**Published:** 2026-06-29

**Authors:** June-Yung Kim, Jeoung Min Lee, Yangjin Park, Heekyung Lee, Jaegoo Lee

**Affiliations:** 1 Department of Social Work, College of Nursing and Professional Disciplines, University of North Dakota, Grand Forks, ND USA; 2 School of Education, Social Work and Psychological Sciences, University of Missouri-Kansas City, Kansas City, MO USA; 3 The University of Texas at Arlington School of Social Work, Arlington, TX USA; 4 Department of Special Education, Rehabilitation & Counseling, California State University-San Bernardino, San Bernardino, CA USA; 5 School of Social Work, College of Health Sciences, Jackson State University, Jackson, USA

**Keywords:** Adverse childhood experiences (ACEs), Flourishing, Religious beliefs/spirituality, Perceived social support, College students, Research domain criteria (RDoC)

## Abstract

Given the increasing focus on positive outcomes after having experienced adverse childhood experiences (ACEs), continued understanding of protective mechanisms is essential. This study examined the role of a Research Domain Criteria (RDoC)-informed construct of the positive valence system (religious beliefs/spirituality) as a mediator via which ACEs are associated with flourishing among emerging adult college students. Further, the moderating role of an RDoC social process systems construct (social support) was simultaneously assessed in the direct association between ACEs and flourishing and the association between religious beliefs/spirituality and flourishing. Participants were 475 U.S. college students in the emerging adulthood period (ages 18–29). The moderated mediation analysis indicated that ACEs were indirectly associated with flourishing through religious beliefs/spirituality (*b* = –0.19, *Boot SE* = 0.05, 95% CI = –0.30, –0.09), adjusting for covariates. Social support significantly moderated the direct association between ACEs and flourishing (*b* = 0.02, *SE* = 0.01, 95% CI = 0.01, 0.04) but not the association between religious beliefs/spirituality and flourishing (*b* = –0.01, *SE* = 0.00, 95% CI = –0.02, 0.00). Specifically, the negative association between ACEs and flourishing was amplified among students with lower (*b* = –0.93, *SE* = 0.19, 95% CI = –1.30, –0.55) and moderate levels of social support (*b* = –0.57, *SE* = 0.14, 95% CI = –0.84, –0.29). However, among those with higher levels of social support, the negative association between ACEs and flourishing became non-significant (*b* = –0.21, *SE* = 0.20, 95% CI = –0.59, 0.18). Campus interventions that foster religious/spiritual engagement and meaning-making while strengthening social support networks, may enhance flourishing among emerging adult college students exposed to ACEs.

## Introduction

Adverse Childhood Experiences (ACEs; Felitti et al., 1998), involving childhood maltreatment and household dysfunction, have drawn considerable attention from public health. Systematic and meta-analytic reviews of more than two decades of empirical evidence have demonstrated ACEs as significant antecedents related to compromised health, development, and functioning throughout the life span (Hughes et al., 2017). Meanwhile, emerging adulthood, typically spanning from ages 18 to 29 (Arnett et al., 2014), is characterized by significant transitions, including individuation from families of origin and establishment of relationships and educational and employment foundations, encompassing diverse developmental paths from college enrollment and workforce entry to vocational training and gap year exploration (Arnett, 2000). For college students navigating this developmental period within academic settings, ACEs are documented to be critical barriers that may undermine the relational, educational, and psychosocial development (Gresham & Karatekin, 2023). The harms associated with ACEs are particularly concerning, with an estimated national rate of 60 to 85% of emerging adults reporting exposure to at least one form of ACEs (Centers for Disease Control and Prevention, 2023). Given that outcomes may depend on environmental factors working in tandem with early toxic stress exposure (Herman-Smith, 2013), it is crucial to investigate the mechanisms through which emerging adult college students can achieve flourishing following ACEs exposure.

Flourishing is defined as “a multidimensional construct that includes positive psychological characteristics that contribute to feeling content and functioning successfully in life (VanderWeele, 2017).” As flourishing encompasses several aspects of human life, including, but not limited to, emotional, psychological, and social components (Keyes, 2002), it reflects the full range of human functioning. Flourishing, as an optimal overall well-being outcome (VanderWeele et al., 2019), has recently gained interest in the field of trauma research, demonstrating its negative associations with higher levels of exposure to ACEs (Wang et al., 2022; Whitaker et al., 2020). The developmental resilience perspective (Masten, 2018) suggests that, even when individuals are exposed to early life adversities, they can become resilient via certain factors that operate at the individual (e.g., personal control, motivation), relational (e.g., parenting competence, peer relationships), and social levels (e.g., safe neighborhoods, social connections; Yoon et al., 2021). To elucidate the mechanisms through which emerging adult college students may achieve flourishing in the context of ACEs, this study focused on religious beliefs/spirituality and perceived social support. These internal assets and external resources, which align with the positive valence and social processes systems of the Research Domain Criteria (RDoC; National Institute of Mental Health [NIMH], n.d), are posited to empower individuals to overcome the adverse impact of ACEs (Chi et al., 2023; Meng et al., 2018; NIMH, n.d.). Since achieving flourishing during emerging adulthood may pave the way for a prospective future (Padilla-Walker & Nelson, 2017), elucidating the mechanisms that help explain the association between ACEs and flourishing could inform targeted interventions.

### RDoC-Informed Mechanisms

The RDoC guides a rigorous assessment of protective and risk processes organized around major domains of functioning, such as systems of positive valence (i.e., processes that respond to positive motivational contexts) and social processes (i.e., processes that respond to various interpersonal settings; NIMH, n.d; Kim et al., 2024, 2025). Religious beliefs/spirituality and perceived social support are self-reported constructs of two RDoC domains, i.e., the positive valence and social processes systems (NIMH, n.d.; Park & Folkman, 1997), which have practical implementation in establishing an evidence-based preventive protocol in community settings, such as a campus context, aimed at effectively promoting flourishing among college students.

#### Religious Beliefs/Spirituality as Positive Valence Systems Construct

Religiosity includes personal belief in God or a higher power and commitment to a religious organization (Zinnbauer et al., 1997), while spirituality involves seeking ultimate purpose, meaning, and the sacred (Puchalski et al., 2014). Due to their conceptual overlap, the terms are often used interchangeably (Zimmer et al., 2016). Among the RDoC positive valence system constructs, religious beliefs/spirituality stand out as salient factors, particularly as a reward valuation construct that involves context-specific evaluation of the probabilities of prospective outcomes (NIMH, n.d). Religious beliefs/spirituality can function as a positive resource by providing a sense of coherence and meaning, functioning as intrinsic motivation and offering rewarding experiences through appraisal of situational meaning or specific events (Park & Folkman, 1997), which help emerging adult college students stay focused on their goals, meaning making, and generativity (Brewer-Smyth & Koenig, 2014; Park, 2022).

Research has consistently shown that individuals exposed to higher levels of ACEs reported lower levels of religious beliefs/spirituality (Santoro et al., 2016; Walker et al., 2009). Park and Folkman (1997) explained that over time, childhood experiences shape one’s overall perspective, ultimately impacting religious and spiritual views as a critical part of global meaning. Notably, this relationship may be complicated by spiritual struggles, such as tensions, strains, and conflicts regarding sacred matters, that can emerge particularly among trauma-exposed individuals (Pargament et al., 2011). Individuals exposed to early life stress may face unique challenges in maintaining positive religious beliefs/spirituality due to existential questions about suffering and divine benevolence (Altmaier, 2019; Walker et al., 2009). McCormick et al. (2017) specifically found that religious/spiritual struggles served as a risk process through which ACEs were associated with mental health symptoms among college students.

Despite these potential struggles, emerging evidence suggests that positive religious beliefs/spirituality, characterized by religious salience, perceived divine care, and sacred purpose, can serve as robust protective factors (Altmaier, 2019; Cotton et al., 2010). Emerging adults with higher levels of positive religious beliefs/spirituality are more likely to flourish in multiple ways with healthy attitudes, behaviors, and self-esteem (Nelson & Padilla-Walker, 2013), even after exposure to childhood adversities (Howell & Miller-Graff, 2014). Among college students, spiritual well-being was associated with psychological health (Leung & Pong, 2021), and those who felt close with God reported increased well-being and a sense of belongingness (Gilbertson et al., 2022). Additionally, recent review studies identified religiosity/spirituality as a protective mechanism that leads to higher levels of resilience in the context of childhood maltreatment (Meng et al., 2018; Yoon et al., 2021). Given this evidence, positive religious beliefs/spirituality may serve as a protective pathway through which the ACEs are associated with flourishing among emerging adult college students, warranting empirical investigation.

#### Social Support as Social Processes System Construct

Defined as the perception of being cared for and receiving assistance from significant others within an interpersonal network of mutual trust (Thoits, 1995), social support can be regarded as an affiliation and attachment, and social communication constructs within the RDoC social processes system domain (NIMH, n.d.). By reinforcing emotional connections and facilitating social communication, perceived social support, as a protective moderator, buffers the detrimental effects of early life adversity (Evans et al., 2013), while strengthening the sense of coherence and meaning in life derived from religious beliefs/spirituality (Fatima et al., 2018). Consistently, prior literature has provided evidence for the protective moderating role of perceived social support which mitigates the adverse effects of ACEs (Chi et al., 2023; Meng et al., 2018), particularly on mental health outcomes (Kealy et al., 2020).

Few empirical studies have specifically examined the moderating role of social support in the association between religious beliefs/spirituality and flourishing among emerging adults exposed to ACEs. However, Upenieks (2022) raised an interesting point that roles of practicing religious beliefs/spirituality, apart from feeling close to God, include providing multiple sets of mentors, resources for support, and engagement in fellowship that is based on common beliefs. Similarly, VanderWeele (2017) suggested higher levels of religious beliefs/spirituality may lead to flourishing across various aspects of life, particularly influenced by social interactions and support from communal religious participation. Thus, social support may serve as a protective mechanism in the complex associations between ACEs, religious beliefs/spirituality, and flourishing. Particularly, social support may not only moderate the adverse association of ACEs with college students’ flourishing but also amplify the positive association between religious beliefs/spirituality and flourishing.

### Present Study

This study aimed to examine the association between ACEs and flourishing among emerging adult college students, while assessing the mechanisms of the RDoC positive valence systems (i.e., religious beliefs/spirituality) and social processes systems domains (i.e., perceived social support). Religious beliefs/spirituality and perceived social support provide developmentally significant internal and external resources that are crucial for college students as they navigate critical demands of emerging adulthood, characterized as financial and individual independence and establishing educational foundations for long-term life goals (Arnett, 2000, 2014; NIMH, n.d.). The specific research questions are: (1) Do religious beliefs/spirituality mediate the association between ACEs and flourishing among college students? (2) Does perceived social support moderate the association between ACEs and flourishing among college students? and (3) Does the association between religious beliefs/spirituality and flourishing differ by the levels of perceived social support among college students?

**Fig. 1:**
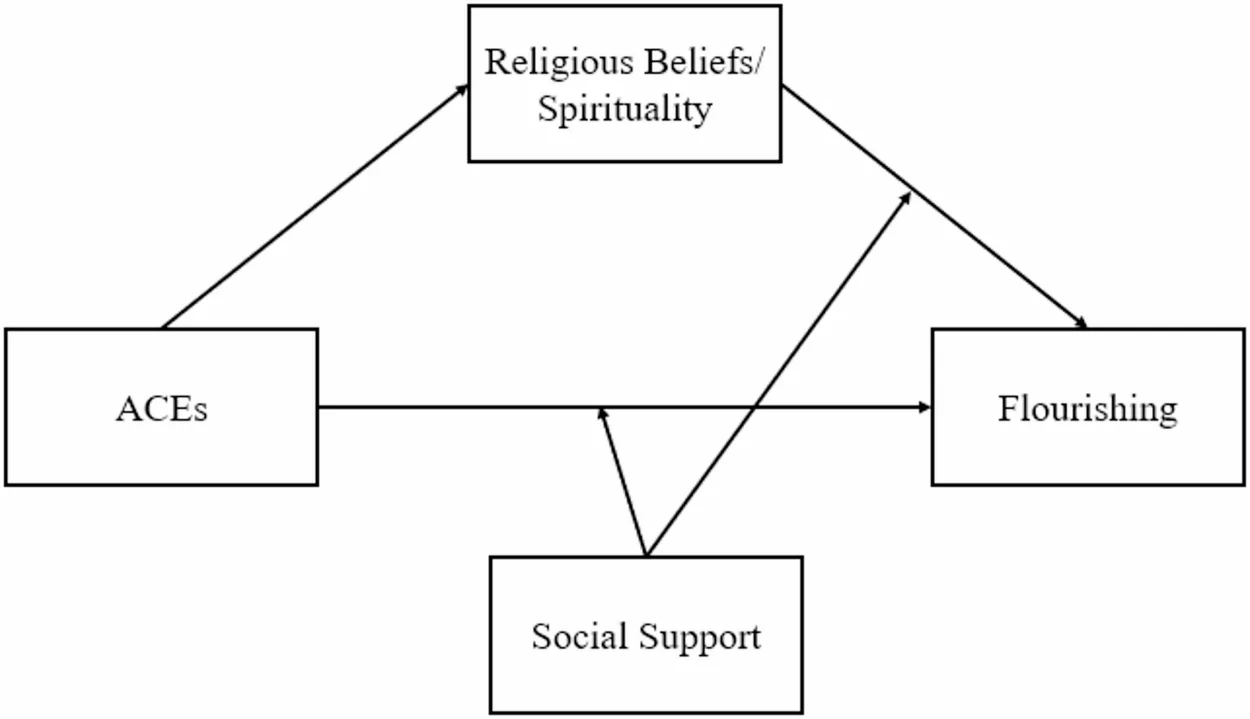
Conceptual model of the association between adverse childhood experiences (ACEs) and flourishing among college students: The mediating role of religious beliefs/spirituality and the moderating role of social support

It was hypothesized emerging adult college students with greater exposure to ACEs would have lower religious beliefs/spirituality, which would in turn be associated with lower flourishing, after adjusting for covariates. We also hypothesized that the association between ACEs and flourishing would be dependent on the levels of perceived social support. Lastly, we hypothesized that the association between religious beliefs/spirituality and flourishing would differ across levels of perceived social support. Figure 1 displays our conceptual model.

This study focused on college students as they represent a substantial and accessible subset of emerging adults (approximately 40% nationally; National Center for Education Statistics, 2023). Examining flourishing among emerging adult college students in the context of ACEs is significant as it shifts the focus from surviving adversity to achieving optimal functioning, taking a more holistic approach to the promotion of health and well-being beyond morbidity and mortality (Wang et al., 2022). Further enhancing our understanding about how religious beliefs/spirituality and perceived social support function as protective mechanisms, which are modifiable mediator and moderator, will inform the design of targeted interventions and support measures to promote flourishing during emerging adulthood.

## Methods

### Study Participants and Procedure

This study was designed as cross-sectional, non-experimental survey research. Potential participants were recruited at the researchers’ affiliated institutions across the midwestern, southeastern, and southern U.S. regions. The study commenced after obtaining approval from the Institutional Review Board. In the informed consent, participants were provided with the information regarding the study, confidentiality explanation, participants’ eligibility criteria (e.g., age 18 years or older), and voluntary nature of the study participation. A self-administered online survey was developed using Qualtrics. Researchers dispatched emails with the study flyer, informed consent, and survey link to students at affiliated universities. Data were collected from participants who agreed and voluntarily participated from November 2022 to January 2023.

This study included 475 emerging adult college students. Of the 878 students who initially accessed the online survey, 578 (65.8%) completed the survey protocols. Excluded were 28 participants aged 30 and above per our theoretical framework of emerging adulthood (Arnett et al., 2014) and 75 with missing data on our key study variables, yielding a final analytic sample of 475. Comparison analyses revealed that those excluded due to missing data were more likely to be White and heterosexual (*ps* < .05), compared to the included. No other significant differences were noted, suggesting data were missing at random (MAR) for our focal study constructs. The participating college students’ ages ranged from 18 to 29 years (*M* = 21.32, *SD* = 2.36). The sample included 136 males (28.6%) and 339 females (71.4%). Participants identified as 304 (64%) White, 65 (13.7%) Black, 50 (10.5%) Hispanic, 53 (11.2%) Asian/Pacific Islander, and 40 (8.4%) others, including American Indian or Alaska Native, mixed races, and other groups; participants could choose multiple responses for this question. Approximately 70.5% (*n* = 335) of the participants identified as heterosexual, while the remaining (29.5%; *n* = 140) identified as sexual minorities.

### Measures

#### Flourishing

Flourishing was assessed using the Flourishing Scale (Diener et al., 2010). The scale was composed of eight items (e.g., “I lead a purposeful and meaningful life,” “My social relationships are supportive and rewarding,” and “I am optimistic about my future.”) with responses measured on a 7-point Likert-type scale, ranging from 1 = *strongly disagree* to 7 = *strongly agree.* The scores were computed by summing the eight items, with higher scores indicating greater flourishing. The coefficient alpha for this scale in the current sample was 0.90.

#### Adverse Childhood Experiences (ACEs)

ACEs were assessed using the Adverse Childhood Experiences International Questionnaire (ACE-IQ; World Health Organization, n.d.). It consisted of 21 items that retrospectively cover different types of adverse childhood experiences across 10 categories: physical (*k* = 2), emotional (*k* = 2), and sexual abuse (*k* = 4); physical (*k* = 3) and emotional neglect (*k* = 2); and family dysfunction, which included household violence (*k* = 3), mental health issues (*k* = 1), incarceration (*k* = 1), substance use (*k* = 1), and parental separation (*k* = 2) experienced prior to age 18. Response options were coded: 0 = *“not exposed*,” and 1 = “*exposed*.” The scores were then combined to form the total ACEs score, representing the number of categories where adverse experiences occurred (ranging from 0 to 10). A cumulative score of four or more indicates a clinical range (Merrick et al., 2024) and was used in this study to provide descriptive context regarding sample severity. In our inferential analyses, however, the continuous total ACEs score was utilized to maximize statistical sensitivity. The coefficient alpha for the current sample was 0.77.

#### Religious Beliefs/Spirituality

Religious beliefs/spirituality were measured using three items that assessed participants’ current religious and spiritual salience, practice, and belief (Cotton et al., 2010). Items were “How important are your religious beliefs/spirituality and practice to you?”, “God cares about even my smallest problems,” and “God has plans for my life.” Responses were measured on a 5-point Likert-type scale, ranging from 1 = *none at all* to 5 = *a great deal*. The scores were computed by summing the three items. The coefficient alpha for the current sample was 0.96.

#### Perceived Social Support

Perceived social support was measured via the Multidimensional Scale of Perceived Social Support (MSPSS; Zimet et al., 1990), which consisted of 12 items asking about the support of family, friends, and special persons. Item examples include, “There is a special person who is around when I am in need,” “My friends really try to help me,” and “I have friends with whom I can share my joys and sorrows.” Responses were measured on a 7-point Likert-type scale, ranging from 1 = *very strongly disagree* to 7 = *very strongly agree*. The scores were computed by summing the items. The coefficient alpha for this scale in the current sample was 0.94.

#### Covariates

Covariates included age, biological sex (male coded as 1), and sexual identity (heterosexual coded as 1). Participants could select multiple racial/ethnic identities; thus, each identity was coded as a separate binary indicator variable: White, Black, Hispanic, Asian, and others, including American Indian or Alaska Native, mixed races, and others.

### Data Analysis

Descriptive statistics and bivariate correlation analyses were conducted to examine the distributional characteristics of the study variables and their bivariate associations. To test our study hypotheses, the mediation and conditional process modeling were conducted using Models 4 and 15 from the PROCESS Macro 4.2 (Hayes, 2022), while adjusting for covariates, including age, biological sex, sexual identity, and race. The indirect effects of religious beliefs/spirituality on the association between ACEs and flourishing were examined, using Model 4 (hypothesis 1). Model 15 was used to simultaneously examine whether perceived social support moderated the association between ACEs and flourishing (hypothesis 2), and whether perceived social support moderated the association between religious beliefs/spirituality and flourishing (hypothesis 3); 95% confidence intervals for the moderated mediation effects were computed using nonparametric bootstrap resampling with 5,000 bootstrap draws (Hayes & Scharkow, 2013). Bootstrap confidence intervals that do not contain zero indicate statistical significance of indirect effects.

**Fig. 2:**
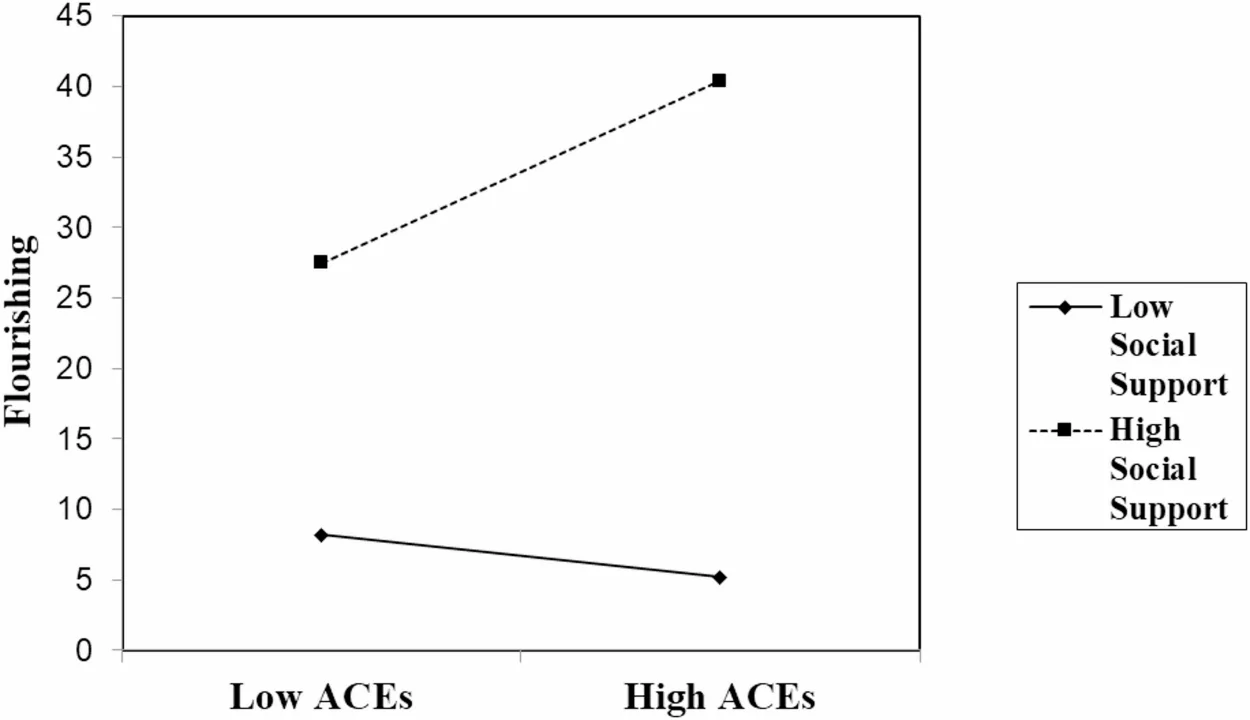
Adjusted predicted mean values of flourishing by adverse childhood experiences (ACEs) and perceived social support

In the event of a significant moderating effect, the PROCESS Model 15 generated a post hoc decomposition analysis results for the interaction to probe the statistical significance of the regression slopes of ACEs on flourishing at low social support (one standard deviation below the mean; Table 3), moderate social support (the mean), and high social support (one standard deviation above the mean). We further plotted a graph of regression lines adjusted for covariates for low and high values of social support to better communicate the nature of the interaction (Warner, 2012; Fig. 2). All continuous variables were mean centered prior to the multivariate analysis to reduce the multicollinearity and increase the interpretability of the estimates. Two-sided *p* <.05 indicates statistical significance. All analyses were conducted using SPSS 29.0.

## Results

Table 1 presents descriptives of study variables, along with zero-order correlations. About 46% of our participants (*n* = 218) reported a history of physical abuse; 59% (*n* = 281) emotional abuse; 31% (*n* = 147) sexual abuse; 26% (*n* = 124) physical neglect; 95% (*n* = 451) emotional neglect; 60% (*n* = 283) household violence; 38% (*n* = 180) mental health issues; 24% (*n* = 114) substance use; and 37% (*n* = 177) parental separation. The ACEs scores ranged from 0 to 10 with 0.2% reporting 0 and 44% meeting the clinical threshold of four or more ACEs.

**Table 1:** Descriptive statistics and correlations among the study variable

	*M (SD)*	*n *(%)	1	2	3	4	5	6	7	8	9	10	11	12
1. ACEs	4.30(2.5)		–											
2. Flourishing	42.37(8.9)		–.35^***^	–										
3. Religious beliefs/spirituality	8.61(4.7)		–.28^***^	.31^***^	–									
4. Perceived social support	63.24(16.2)		–.23^***^	.55^***^	.08	–								
5. Ages	21.32(2.4)		.13^**^	–.15^**^	–.10^*^	–.04	–							
6. White		304(64)	.09	.05	–.17^***^	.10^*^	–.04	–						
7. Black		65(13.7)	.00	.07	.33^***^	–.07	–.04	–.45^***^	–					
8. Hispanic		50(10.5)	–.01	.00	.06	.02	.10^*^	–.36^***^	–.14^**^	–				
9. Asian		53(11.2)	–.10^*^	–.09^*^	–.18^***^	–.04	.02	–.40^***^	–.14^**^	–.12^**^	–			
10. Others		40(8.4)	.08	–.14^**^	.05	–.14^**^	.10^*^	–.17^***^	–.03	–.03	–.08	–		
11. Biological sex			.15^**^	.06	.05	.12^*^	–.12^**^	.05	.02	–.04	–.10^*^	.01	–	
Male		136(28.6)												
Female		339(71.4)												
12. Sexual identity			–.24^***^	.13^**^	.31^***^	–.02	.02	–.08	.07	.03	.04	.03	–.16^**^	–
Homosexual		140(29.5)												
Heterosexual		335(70.5)												

^*^p <.05 ^**^p <.01 ^***^ *p* <.001

ACEs = Adverse childhood experiences; Others = American Indian or Alaska Native, Mixed races, and Others.

Bivariate correlations among study variables ranged from –0.45 to 0.55, indicating collinearity was not an issue. ACEs were negatively correlated with flourishing (*r* = −.35, *p* <.001), religious beliefs/spirituality (*r* = −.28, *p* <.001), and perceived social support (*r* = −.23, *p* <.001). Flourishing was positively correlated with religious beliefs/spirituality (*r* = .31, *p* <.001), and perceived social support (*r* = .55, *p* <.001). Age was positively correlated with ACEs (*r* = .13, *p* <.01) and negatively correlated with flourishing (*r* = −.15, *p* <.01) and religious beliefs/spirituality (*r* = −.10, *p* <.05). Black participants reported higher levels of religious beliefs/spirituality compared to other groups (*r* = .33, *p* <.001). Heterosexual-identifying participants reported lower ACEs exposure (*r* = −.24, *p* <.001) and higher religious beliefs/spirituality (*r* = .31, *p* <.001) and flourishing (*r* = .13, *p* <.01), compared to sexual minority participants. Females reported higher ACEs exposure than males (*r* = .15, *p* <.01).

### Mediating Effects of Religious Beliefs/Spirituality

Table 2 reports the adjusted estimated associations among ACEs, religious beliefs/spirituality, and flourishing. ACEs were negatively associated with religious beliefs/spirituality (*b* = −0.46, *p* <.001), which in turn was positively associated with flourishing (*b* = 0.40, *p* <.001). Table 2 also shows the bootstrap results of the indirect effects of ACEs on flourishing through religious beliefs/spirituality. ACEs were negatively associated with flourishing (*b* = −1.02, *Boot SE* = 0.16, 95% CI [–1.34, –0.71]). The indirect effect of religious beliefs/spirituality (*b* = –0.19, *Boot SE* = 0.05, 95% CI [–0.30, –0.09]) was significant, as the bootstrapped confidence interval did not include zero, thus supporting our first hypothesis.

**Table 2:** Mediation analysis: Path coefficients and indirect effects

Path Coefficients				
Variables	Model 1 (Religious beliefs/spirituality)		Model 2 (Flourishing)	
	*b*	*t*	*b*	*t*
Constant	−56.22^***^	−26.83	23.12^***^	3.48
ACEs	−0.46^***^	−5.95	−1.02^***^	−6.37
Religious beliefs/spirituality	–	–	0.40^***^	4.35
Ages	−0.46	−1.46	−0.25	−1.56
White	−0.12	−0.26	1.87	1.25
Black	4.04^***^	4.76	0.10	0.58
Hispanic	1.22	1.40	0.87	0.50
Asian	−2.21^*^	−2.47	−0.97	−0.54
Others	1.04	1.38	−3.31	−2.22
Female	2.60^***^	6.14	0.40	0.46
Heterosexual identity	1.08^*^	2.57	1.63	1.93
*R* ^2^	0.29		0.21	
*F*	21.38^***^		12.34^***^	

Indirect Effects (Bootstrap)				
Path	*b*	*Boot SE*	*95% CI*	
Total Effect: ACEs → Flourishing	–1.21	0.16	[–1.52, –0.90]	
Direct Effect: ACEs → Flourishing	–1.02	0.16	[–1.34, –0.71]	
Indirect Effect: ACEs → Religious beliefs/Spirituality → Flourishing	–0.19	0.05	[–0.30, –0.09]	

^*^p <.05 ^**^p <.01 ^***^ *p* <.001

Unstandardized coefficients are reported.

ACEs = Adverse childhood experiences; CI = confidence interval; Others = American Indian or Alaska Native, Mixed races, and others.

Black participants reported higher religious beliefs/spirituality (*b* = 4.04, SE = 0.85, 95% CI [2.37, 5.71]), while Asian participants reported lower levels (*b* = − 2.21, SE = 0.89, 95% CI [–3.96, − 0.45]). Female participants (*b* = 2.60, SE = 0.42, 95% CI [1.77, 3.43]) and heterosexual-identifying participants (*b* = 1.08, SE = 0.42, 95% CI [0.25, 1.92]) reported higher religious beliefs/spirituality compared to male and homosexual-identifying participants, respectively.

### Moderating Effects of Perceived Social Support

Table 3 presents the results of the conditional process analysis. After adjusting for the covariates, ACEs were negatively associated with flourishing (*b* = –0.57, *SE* = 0.14, 95% CI [–0.84, –0.29]). The interaction term between ACEs and perceived social support was significantly associated with flourishing (*b* = 0.02, *SE* = 0.01, 95% CI [0.01, 0.04]), indicating that the association between ACEs and flourishing is dependent on the levels of social support, consistent with our second hypothesis. As reported in the conditional direct association between ACEs and flourishing (Fig. 2), among participants with lower (*b* = –0.93, *SE* = 0.19, 95% CI [–1.30, –0.55]) and moderate levels of social support (*b* = –0.57, *SE* = 0.14, 95% CI [–0.84, –0.29]), the negative association between ACEs and flourishing was significant. However, among participants with higher levels of social support, the negative association between ACEs and flourishing was not significant (*b* = –0.21, *SE* = 0.20, 95% CI [–0.59, 0.18]).

**Table 3:** Conditional process analysis: Flourishing as outcome

	*b*	*SE*	*LL 95% CI*	*UL 95% CI*
Constant	4.98	1.41	–1.97	11.93
ACEs	–0.57^***^	0.14	–0.84	–0.29
Religious beliefs/spirituality	0.37^***^	0.08	0.21	0.52
Perceived social support	0.27^***^	0.02	0.23	0.31
ACEs X Perceived social support	0.02^**^	0.01	0.01	0.04
Religious beliefs/spirituality X Perceived social support	–0.01	0.00	–0.02	0.00
Ages	–0.33^*^	0.14	–0.60	–0.06
White	1.39	1.27	–1.10	3.88
Black	1.66	1.46	–1.22	4.54
Hispanic	0.73	1.47	–2.15	3.61
Asian	–0.56	1.52	–3.54	2.42
Others	–1.06	1.28	–3.58	1.45
Females	1.19	0.74	–0.27	2.66
Heterosexual identity	0.28	0.72	–1.14	1.71

Conditional direct effect of ACEs on flourishing				
Lower social support (M – 1SD)	–0.93^***^	0.19	–1.30	–0.55
Moderate social support (Mean)	–0.57^***^	0.14	–0.84	–0.29
Higher social support (M + 1SD)	–0.21	0.20	–0.59	0.18

^*^p <.05 ^**^p <.01 ^***^ *p* <.001

Unstandardized coefficients are reported; bootstrap sample size = 5,000.

ACEs = Adverse childhood experiences; Others = American Indian or Alaska Native, Mixed races, and others; LL = lower limit; UL = upper limit; CI = confidence interval.

The interaction term between religious beliefs/spirituality and social support was not significant (*b* = –0.01, *SE* = 0.00, 95% CI [–0.02, 0.00]). Inconsistent with our third hypothesis, perceived social support did not moderate the association between ACEs and flourishing through religious beliefs/spirituality, indicating that the indirect effect of ACEs on flourishing through religious beliefs/spirituality did not vary across levels of perceived social support. Age was the only covariate that was associated with flourishing (*b* = –0.33, SE = 0.14, 95% CI [–0.60, –0.06]).

## Discussion

This study examined religious beliefs/spirituality as a potential mediator of the association between ACEs and flourishing among college students, while also assessing perceived social support as a potential moderator both in the association between ACEs and flourishing and the association between religious beliefs/spirituality and flourishing. As hypothesized, ACEs were associated with flourishing through religious beliefs/spirituality. Consistent with our second hypothesis, social support moderated the association between ACEs and flourishing. However, social support did not moderate the association between religious beliefs/spirituality and flourishing, refuting our third hypothesis. This study is one of the first that associated ACEs with flourishing among emerging adult college students, while further exploring RDoC-informed mechanisms, a key strength essential for informing evidence-based intervention strategies in community settings aimed at promoting flourishing in college students.

Our findings corroborate existing ACEs literature (e.g., Hughes et al., 2017; Yoon et al., 2021), notably adding to the body of knowledge on resilience in the context of ACEs and protective mechanisms. Our findings extend previous studies on ACEs and flourishing (Crandall et al., 2020; Wang et al., 2022; Whitaker et al., 2020) by examining the significance of religious beliefs/spirituality as a source of coherence and purpose, essential for achieving flourishing among college students in emerging adulthood. Religious beliefs/spirituality may provide a stable and adaptive life narrative, helping college students make sense of their early life adversity in a meaningful way and stay motivated toward their goals (Park & Folkman, 1997). This meaning-making process can reinforce optimism and contribute to a sense of control over one’s future, all of which are critical in navigating the complex developmental transitions of emerging adulthood (Brewer-Smyth & Koenig, 2014). Furthermore, religious and spiritual practices, such as prayer or participation in faith-based communities, may create structured routines that further promote overall well-being (VanderWeele, 2017, 2020).

In our sample, perceived social support moderated the association of ACEs with flourishing, consistent with prior research suggesting a significant role of perceived social support in buffering the adverse impacts of ACEs (Mosley-Johnson et al., 2019). Among emerging adult college students who perceive they do not receive substantial social support from significant others, the adverse association of ACEs and flourishing was amplified. However, those with substantial social support reported a mitigated negative association between ACEs and flourishing, reducing it to a statistically insignificant level. This finding reinforces the protective function of perceived social support as an affiliation and attachment construct within the RDoC social processes system domain (NIMH, n.d.), aligning with previous studies demonstrating its moderating effects on the association between ACEs and mental health outcomes (Chi et al., 2023; Meng et al., 2018).

We further tested a moderating role of perceived social support in the association between religious beliefs/spirituality and flourishing. However, social support was not significant in moderating this association. The current finding may reflect that college students’ intrinsic motivational and reward-based aspects of religious beliefs/spirituality can sufficiently promote flourishing without additional external moderation from social support (Steffen et al., 2017). The features of affiliation and attachment may already be encompassed within practicing religious beliefs/spirituality, thereby independently enhancing flourishing. Alternatively, it is possible that social support may only enhance the positive effects of religious beliefs/spirituality when it comes from individuals who share similar spiritual values and practices (Holt et al., 2018). Future research should further examine these dynamics to determine under what conditions social support strengthens the relationship between religious beliefs/spirituality and flourishing, particularly among emerging adult college students exposed to ACEs.

It is also noteworthy that older age was correlated with higher ACEs exposure alongside lower levels of flourishing and religious beliefs/spirituality, suggesting developmental nuances within the theoretically bounded period of emerging adulthood. While the correlational nature of these associations precludes causal inferences, these patterns align with the characterization of emerging adulthood as a period of instability (Arnett, 2000, 2014); the transition toward the late twenties often involves increased pressure regarding career establishment and independence, which may temporarily tax psychological resources and spiritual engagement compared to younger college students. Recognizing this heterogeneity, the current analysis controlled for age to rigorously isolate the mechanisms of modifiable protective factors independent of these potential developmental variances. Future exploration of age as a potential moderator in these pathways using longitudinal designs could determine whether the protective effects of religious beliefs/spirituality and social support vary across developmental transitions.

Limitations should be considered when interpreting the current findings. First, the generalizability of our findings is limited, as the data were collected at universities in the Midwest region of the U.S. where the majority of the participating emerging adult college students were White. Relatedly, the 65.8% completion rate may have introduced self-selection bias, as students who completed the survey may differ systematically from non-completers in unmeasured ways. Additionally, college students represent only one subset of emerging adults (nearly 40%; NCES, 2023); our findings may not generalize to emerging adults in other developmental contexts, such as those entering the workforce or pursuing vocational training. Second, although the ACEs questionnaire provided a time reference for participants to recall their childhood prior to age 18, the cross-sectional, correlational nature of our data does not reflect causal relationships. In line with this limitation, the use of a retrospective self-report to assess ACEs may be less reliable due to potential memory errors (Reuben et al., 2016). However, retrospective self-reports of early life adversity can often be more valid than prospective measures (Baldwin et al., 2019); how individuals perceive their ACEs may hold greater significance than objective assessments. Third, religious beliefs/spirituality was assessed using a brief three-item measure (Cotton et al., 2010) that, while demonstrating excellent internal consistency (α = 0.96), may not fully capture the multidimensional nature of religious and spiritual experiences. Our measure focused on positive religious/spiritual resources (religious salience, perceived divine care, sacred purpose) but did not assess spiritual struggles—tensions and questions regarding sacred matters that may arise as individuals process adversity (Exline et al., 2014; Pargament et al., 2011). Future research employing more comprehensive assessments of how individuals navigate their faith in the context of early life stress would provide deeper insights into the religious/spiritual pathways to flourishing. Lastly, although the religious beliefs/spirituality component of RDoC positive valence system and the social support component of the RDoC social processes system were carefully assessed as mechanisms informed by prior literature, we could not account for a wide range of factors due to the data availability. Other individual (e.g., personal control, social competence), relational (e.g., caring adults, peer relationship), and social (e.g., school climate, safe neighborhood; Yoon et al., 2021) factors across other domains of RDoC functioning (e.g., cognitive systems, negative valence systems) may also account for emerging adult college students’ achieving flourishing following ACEs, which warrants future examination.

### Practice Implications

Religious beliefs/spirituality provide a worldview that helps individuals find meaning in their experiences, particularly in processing adversity (Gilbertson et al., 2022). Given this, universities could implement faith-based counseling services, mentorship programs, or structured spaces where students can process their struggles in a supportive and growth-oriented context, while respecting individual differences in religious/spiritual practices. These initiatives may help students contextualize life challenges as part of their broader personal and spiritual development. Acknowledging that some individuals may experience spiritual struggles in processing adversity (Exline et al., 2014), interventions should be voluntary and sensitive to individual differences in religious/spiritual practices. Within the broader framework of flourishing interventions, fostering religious/spiritual engagement and meaningful relational connections can be particularly beneficial. Religious service attendance may be incorporated into trauma-informed interventions as a means of expanding students’ social networks and strengthening emotional support systems. Additionally, participation in community organizations or volunteer services may complement these efforts by further broadening relational connections. This comprehensive approach can positively impact other key dimensions of flourishing, such as interpersonal relationships, meaning in life, and character development (Koenig et al., 2012).

Our findings also highlighted the role of social support in mitigating the negative association between ACEs and flourishing. Family members, close friends, and faculty may serve as crucial sources of support for college students (VanderWeele, 2020). Trauma-informed interventions can be designed to empower students, particularly those who experienced early life stress, to actively build and maintain social support networks. Universities can create supportive environments by implementing interventions targeting meaningful social connections (Kobau et al., 2011). Promising models include Well-Being Therapy (Ruini & Fava, 2014) and Positive Psychotherapy (Rashid, 2015), integrable into campus-based initiatives. Curriculum-based interventions, such as elective courses on well-being, have shown promise in improving flourishing (Hirshberg et al., 2022), with topics focusing on relationship-building, self-awareness, and personal growth (Tanase & Dinsmore, 2023). Faculty members can play a pivotal role in delivering these interventions, guiding students toward available resources and fostering a campus culture that meaningfully promotes flourishing (VanderWeele, 2020).

### Conclusion

ACEs were associated with flourishing via religious beliefs/spirituality among emerging adult college students. Social support mitigated the negative association between ACEs and flourishing, although it did not moderate the association between religious beliefs/spirituality and flourishing. Our findings contribute to the ACEs literature by elucidating the complex interplay among ACEs, religious beliefs/spirituality, and social support as important social determinants of flourishing. Campus interventions that foster religious and relational engagement and strengthen social support networks may enhance flourishing among students exposed to ACEs. Continued research on flourishing in later adulthood in the context of ACEs, with rigorous examinations of key mechanisms (e.g., neuroplasticity as cognitive systems construct, Davidson & McEwen, 2012; a safe and stable environment as negative valence systems construct, Blair & Raver, 2015), will advance comprehensive knowledge of promoting flourishing among ACEs-exposed individuals.
